# Mechanical Intelligence in Bone Regeneration: Bridging Material and Cellular Memory for Enhanced Healing

**DOI:** 10.1002/advs.76638

**Published:** 2026-07-16

**Authors:** Jin Tian, Guoyou Huang, Yang Chen, Teng Ma, Bo Cheng, Feng Xu, XiaoKang Li, Pei Yang, Ting Wen

**Affiliations:** ^1^ The Second Affiliated Hospital of Xi'an Jiaotong University Xi'an People's Republic of China; ^2^ Bioinspired Engineering and Biomechanics Center (BEBC) School of Life Science and Technology Xi'an Jiaotong University Xi'an People's Republic of China; ^3^ The Key Laboratory of Biomedical Information Engineering of Ministry of Education School of Life Science and Technology Xi'an Jiaotong University Xi'an People's Republic of China; ^4^ Department of Engineering Mechanics School of Civil Engineering Wuhan University Wuhan People's Republic of China; ^5^ Joint and Foot & Ankle Ward of Orthopedic Center the Second Affiliated Hospital of Xi'an Jiaotong University Xi'an People's Republic of China; ^6^ Severe and Polytrauma Department of Orthopaedic Surgery Honghui Hospital Xi'an Jiaotong University Xi'an People's Republic of China; ^7^ FX Group‐Xi'an Jiaotong University Institute of Life Health Xi'an People's Republic of China; ^8^ Hainan General Hospital Hainan Affiliated Hospital of Hainan Medical University Haikou People's Republic of China

**Keywords:** bone regeneration, mechanotransduction, scaffold, stiffness, stress shielding

## Abstract

Mechanical cues shape bone regeneration, but treatments for delayed union, nonunion, and mechanically mismatched repair often still treat them as static constraints. In this review, we use mechanical intelligence to describe the time‐dependent capacity of biomaterials, cells, and therapeutic devices to store, transform, and transmit mechanical history during repair. Two forms of memory are central to this view. Scaffolds and implants can preserve or release previous mechanical states through relaxation, residual stress, shape recovery, evolving stiffness, and architecture, whereas cells can carry earlier stiffness or loading exposure into later mechanotransduction, lineage commitment, and niche remodeling. The key question is when these two forms of memory meet during healing, from cell recruitment and matrix formation to callus maturation, load sharing, and rehabilitation. Coupling material and cellular memory may help match scaffold mechanics to cellular decision windows and tissue competence, support osteogenesis, reduce maladaptive responses such as stress shielding or fibrosis, and guide stage‐specific scaffolds, adaptive fixation, sensing‐assisted modelling, and mechanically timed rehabilitation for personalized bone repair.

## Graphical abstract

Mechanical intelligence reframes bone regeneration as a time‐dependent coupling between material and cellular memory. Biomaterials store and release mechanical history, while cells retain prior cues and remodel the repair niche. Matching these memories to healing stages may guide stage‐specific scaffolds, sensing‐assisted prediction, mechanical dosing, and clinician‐supervised personalized repair.

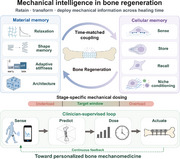

## 1 Introduction

After fracture or bone loss, skeletal tissue can often rebuild its structure and function, but delayed union and nonunion remain major clinical failures. Repair depends on local cells, biochemical signaling, vascularization, immune activity, and the mechanical environment around the defect [1]. The mechanical environment is not simply a boundary condition applied to this process. Bone adapts continually to loading: repeated high‐impact activity in athletes is associated with greater bone mass, whereas microgravity in astronauts leads to marked bone loss [1, 2]. These examples show why mechanotransduction matters for repair, because mechanical cues are converted into biochemical signals that alter cell behavior and tissue formation.

**FIGURE 1:**
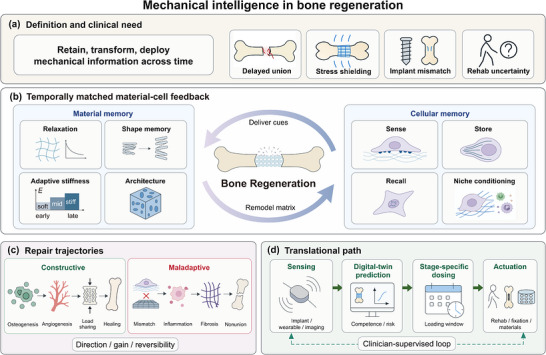
Conceptual framework of mechanical intelligence in bone regeneration. (a) Mechanical intelligence is defined as the ability of a bone‐repair system to use mechanical history to guide later healing. The concept addresses delayed union, stress shielding, implant‐bone mismatch, and uncertainty in rehabilitation timing. (b) At the center of the framework is the timing between material memory and cellular memory. Materials can release or reshape mechanical cues through relaxation, shape recovery, adaptive stiffness, and architecture, whereas cells retain earlier cues through mechanotransduction, cytoskeletal remodeling, matrix deposition, and niche conditioning. (c) If the cue arrives at a compatible healing state, it may support osteogenesis, angiogenesis, integration, and load sharing; if it arrives at the wrong time or magnitude, the response may shift toward inflammation, fibrosis, instability, or nonunion. (d) Clinical deployment requires sensing, patient‐ and construct‐specific modeling, stage‐specific mechanical dosing, and actuation through rehabilitation, fixation, or responsive materials, with clinician‐supervised decision support as the near‐term goal.

Recent studies indicate that implanted materials and host cells can both carry mechanical history after the original cue has changed or disappeared [3]. These observations motivate the framework of mechanical intelligence, which we define here as the capacity of biomaterials, cells, and therapeutic devices to retain, transform, and use mechanical information over time to guide stage‐specific repair (Figure 1). The term builds on the engineering concept of mechanical intelligence, where adaptive structures respond through material and structural design [4]. In bone regeneration, however, the adaptive system also includes living cells that sense mechanical cues, remodel their surroundings, and retain part of that mechanical history.

Mechanical intelligence has three working elements: material mechanical memory, cellular mechanical memory, and material‐cell feedback that is matched to the stage of healing. Material memory denotes stored or recoverable mechanical states, including residual stress, programmed shape, stress relaxation, evolving stiffness, and architectural anisotropy [5, 6, 7, 8, 9]. Cellular memory denotes persistent cytoskeletal, nuclear, transcriptional, or chromatin‐level changes caused by earlier stiffness, loading, or confinement [10, 11, 12, 13, 14, 15]. These memories are most relevant to repair when they arrive at the right biological moment, for example, during cell infiltration, osteogenic commitment, callus mineralization, or progressive load sharing [16, 17, 18, 19, 20]. A bone‐repair system can therefore be judged by whether it stores a mechanical cue, keeps or erases it over a useful time scale, delivers it within an appropriate healing window, and improves outcomes such as callus stiffness, load transfer, osteogenic differentiation, fibrosis control, or nonunion risk. For clinical translation, sensing technologies, digital twins, and artificial intelligence (AI) may then help measure, predict, and apply these mechanical histories in stage‐specific therapy.

Guided by this framework, we focus first on the two memory‐bearing elements of the repair system: biomaterials that retain or release mechanical history through relaxation, residual stress, shape recovery, architecture, and evolving stiffness, and cells that preserve prior mechanical exposure through cytoskeletal, nuclear, transcriptional, and niche‐level responses. We then bring these two elements together at the repair interface, where the key problem is timing: scaffold mechanics must be matched to cellular decision windows, callus maturation, and progressive load sharing. The latter part of the review considers how this coupling can be used clinically, including mechanical‐state sensing, patient‐ and construct‐specific prediction, stage‐specific dosing, rehabilitation, adaptive fixation, responsive materials, and validated decision support.

## 2 Mechanical Memory Encoded in Biomaterials for Bone Healing

Most bone‐repair materials are still selected primarily for initial stiffness, strength, porosity, and degradation rate. Material mechanical memory adds a temporal dimension to this design problem. A scaffold or implant may preserve residual stress, a temporary geometry, a relaxation state, or a preferred architecture after fabrication, implantation, or loading, and these stored states can later alter the mechanical environment experienced by cells. In this review, material mechanical memory refers to such retained mechanical states and to their release, recovery, or modification during repair. The main strategies include viscoelastic and plastic deformation, residual stress and shape‐memory behavior, dynamic modulus or adaptive stiffness, and micro‐/nano‐architectural patterning.

### 2.1 Viscoelastic and Plastic Deformation

**FIGURE 2:**
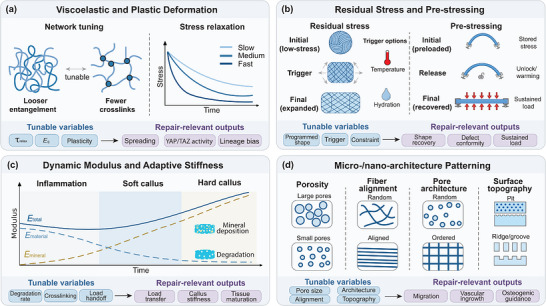
Material mechanical memory strategies for time‐dependent control of the bone repair microenvironment. (a) Viscoelastic or plastic scaffolds can carry loading history through relaxation, creep, chain rearrangement, or crosslinking, which can alter cell spreading and YAP/TAZ‐related lineage responses. (b) Residual stress and programmed temporary shapes store mechanical energy before implantation and release it after hydration, temperature change, or removal of constraint. (c) During repair, scaffold stiffness may increase, decrease, or redistribute load as degradation, mineralization, or secondary crosslinking proceeds; the curve shown is an example rather than a universal trajectory. (d) Pore geometry, fiber alignment, anisotropy, and surface topography guide migration, vascular ingrowth, and osteogenic differentiation through spatial mechanical cues.

Viscoelastic biomaterials demonstrate time‐dependent stress relaxation and creep phenomena, thereby retaining a record of prior strain or stress histories (Figure 2a). Unlike purely elastic materials, viscoelastic or plastic scaffolds can dissipate mechanical loads gradually. This property can be tuned and may influence cellular mechanotransduction pathways, thereby affecting cell behavior and lineage commitment [5, 21]. Mechanical priming on stiff matrices can also create a dose‐dependent cellular response: inhuman mesenchymal stem cells (hMSCs), sufficiently prolonged exposure produced persistent activation of Yes‐associated protein (YAP), transcriptional coactivator with PDZ‐binding motif (TAZ), and runt‐related transcription factor 2 (RUNX2), together with an osteogenic bias after matrix softening [10]. By contrast, viscoelastic alginate hydrogels with rapid stress relaxation preserved the self‐renewal capacity of mesenchymal stem cells (MSCs) and supported their proliferation [22]. Matrix stiffness and viscoelasticity also act together in regulating lineage commitment; MSC osteogenesis was enhanced in hydrogels with an initial elastic modulus of approximately 17 kPa when accompanied by pronounced stress dissipation [23].

### 2.2 Residual Stress and Pre‐Stressing

Materials can be fabricated or pre‐conditioned with internal stresses that serve as stored mechanical cues. Upon deployment, these stresses can be released in a controlled manner to perform work or alter material shape or mechanics (Figure 2b). Shape‐memory systems can be programmed into a temporary configuration and recover through thermal, hydration‐, light‐, or stress‐triggered phase or network transitions [24, 25]. In bone repair, shape‐memory scaffolds can support minimally invasive insertion followed by in situ expansion and defect conformity. A water‐responsive silk‐fibroin/magnesium scaffold, for example, recovered its programmed shape after contact with body fluid and fitted irregular cranial defects while supporting bone regeneration [6]. Nickel–titanium (NiTi) implants provide a complementary mechanism: recovery toward the austenitic shape at body temperature can generate sustained bending or compression that alters bone modelling or fixation stiffness [25, 26, 27]. These examples show how residual stress and phase transitions can generate time‐dependent mechanical cues, although force magnitude and duration remain device‐specific.

### 2.3 Dynamic Modulus and Adaptive Stiffness

Beyond transient shape changes, biomaterials can be designed so that their mechanical properties evolve during tissue healing (Figure 2c), including stiffening through secondary crosslinking or mineral deposition and softening through degradation [28, 29]. Rather than following a universal modulus trajectory, dynamic systems can soften, stiffen, or redistribute load over days to weeks, and their biological effect depends on the timing and dimensional context of the transition [7, 30]. In a sequentially crosslinked three‐dimensional hydrogel, cells were first allowed to spread and expand before light‐triggered secondary stiffening; later stiffening enhanced osteogenic matrix secretion, YAP/TAZ localization, and bone remodeling in vivo [31]. A complementary macroporous protein‐based hydrogel used rigid fiber‐coated pore shells to provide sustained local mechanical cues while the bulk material degraded in partial synchrony with new tissue deposition [7]. Such platforms therefore implement a tunable mechanical schedule, but the appropriate schedule must be defined for each defect, cell state, and loading environment.

### 2.4 Micro‐/Nano‐Architectural Patterning

Scaffold architecture, including fiber orientation, pore connectivity, anisotropy, and surface topography (Figure 2d), can encode spatial mechanical guidance. Hierarchical collagen organization and mineral association, for example, can influence bone‐graft performance and regeneration [32]. Static patterns are not mechanical memory by themselves, but responsive polymers can reorganize surface features on demand, thereby changing cell adhesion and fate [33]. Stress‐induced martensitic transformation, twinning, or amorphization can also store deformation history at the microstructural level, although this remains primarily a material mechanism rather than a validated bone‐regeneration memory process [8]. Mechanical metamaterials provide a further route to tune scaffold‐level strain fields and load transfer [9, 34]. Architectural guidance should nevertheless be bounded because excessive anisotropy or local stress concentration may impair interface stability.

These findings suggest that scaffold design should be considered in temporal rather than static terms. A scaffold does not need to perform a single function throughout healing; its mechanical role may shift as repair progresses through different stages. Materials with mechanical memory make this possible. Their shape, internal architecture, or stiffness can be programmed to reshape mechanical cues, redistribute strain, or change over time [6, 7, 9, 30, 31]. In the early stage of repair, a softer environment may help cells enter and grow. In later stages, a stiffer structure may help tissue mature and support loading. The main material memory strategies, their mechanisms, advantages, limitations, and representative applications are summarized in Table 1. This leads to a related question. If materials can keep part of their mechanical history, can bone‐repairing cells also keep that history? And does that history affect how the cells respond later?

**TABLE 1:** Comparison of material mechanical memory strategies for bone regeneration.

Strategy	Mechanism	Advantages	Limitations	Representative use
Viscoelastic/plastic deformation	Time‐dependent stress relaxation, creep, and dissipation of prior strain or stress history.	Can tune cellular spreading, YAP/TAZ activity, proliferation, and lineage bias while reducing abrupt mechanical mismatch.	Difficult to define a universal relaxation window; excessive compliance may impair load transfer.	Stress‐relaxing hydrogels and viscoelastic scaffolds for MSC expansion, infiltration, and osteogenic induction [5, 10, 21, 22, 23].
Residual stress/shape‐memory behavior	Pre‐stored stress or temporary geometry is released by temperature, hydration, light, or phase transition.	Supports minimally invasive deployment, self‐fitting, local compression, and defect conformity.	Triggering conditions, recovery force, fatigue, and long‐term interface stability require careful control.	Shape‐memory polymer scaffolds, NiTi fixation elements, and self‐expanding constructs [6, 25, 26, 27].
Dynamic modulus/adaptive stiffness	Material stiffness changes over time through degradation, secondary crosslinking, mineralization, or programable architecture.	Allows scaffold mechanics to be matched to inflammation, callus formation, mineralization, and remodeling stages.	Poorly timed stiffening or softening can cause stress shielding, delayed maturation, or nonunion.	Time‐programmed hydrogels, two‐stage scaffolds, and adjustable fixation systems [7, 28, 29, 30, 31].
Micro‐/nano‐architecture patterning	Porosity, fiber alignment, anisotropy, and topography encode spatial mechanical guidance.	Guides cell migration, alignment, vascular ingrowth, and matrix orientation.	Excessive anisotropy or stress concentration may promote fibrotic alignment or interface failure.	Electrospun scaffolds, porous architectures, microtopographic surfaces, and mechanical metamaterials [8, 9, 32, 33, 34].

## 3 Mechanical Memory Retained by Cells During Bone Regeneration

**FIGURE 3:**
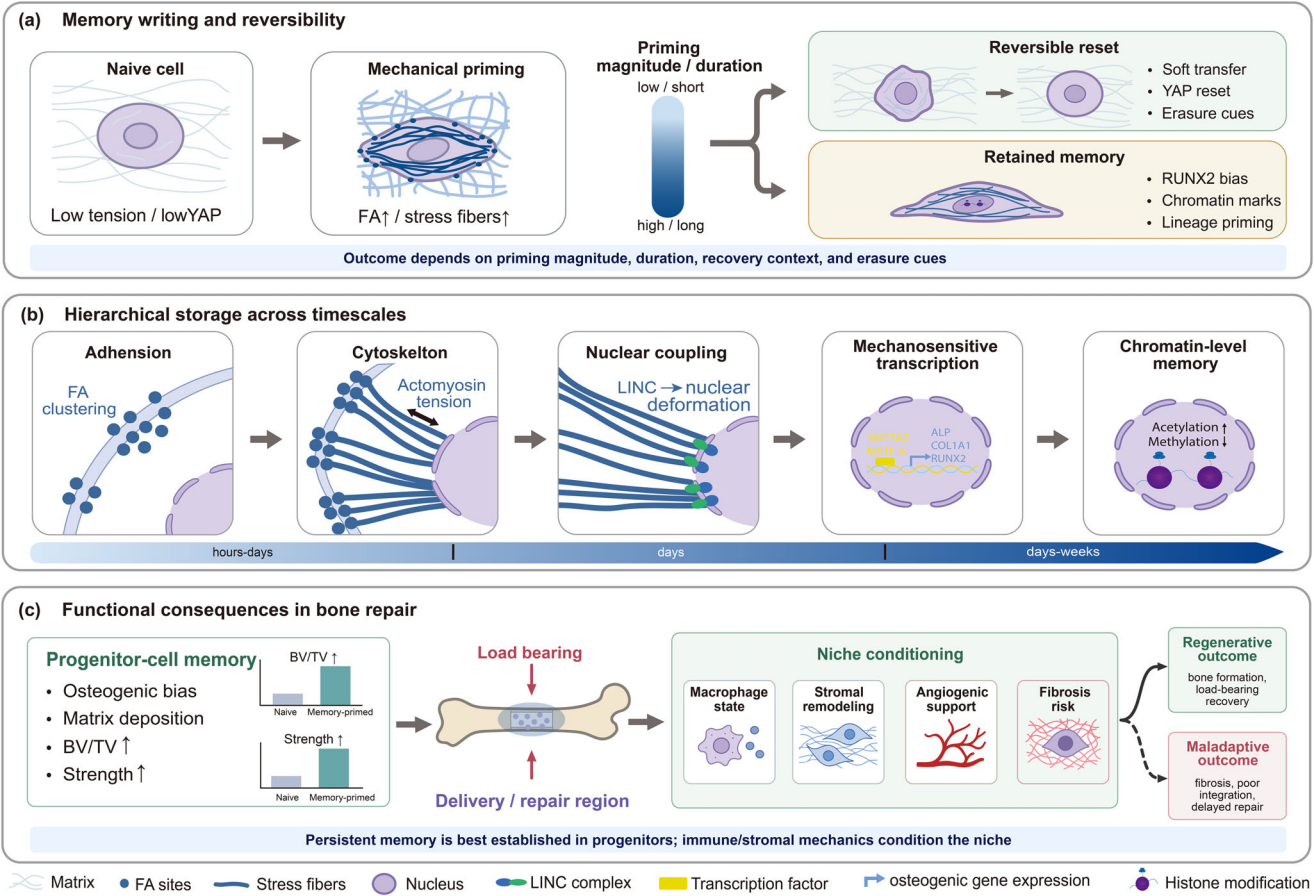
Cellular mechanical memory across timescales and regenerative niches. (a) Mechanical priming can leave a cellular trace through focal adhesion maturation, stress‐fiber formation, actomyosin tension, and YAP/TAZ activity. Short or weak priming may fade after transfer to a softer or competing adhesive environment, whereas longer or stronger exposure can preserve RUNX2 bias, chromatin marks, or lineage priming. (b) Memory‐related changes occur on different timescales, from cytoskeletal remodeling over hours to days to LINC‐mediated nuclear deformation, mechanosensitive transcription, and chromatin‐level regulation over longer periods. (c) In bone repair, the strongest evidence for persistent mechanical memory comes from progenitor cells. Immune and stromal mechanosensing may condition the regenerative niche by shaping macrophage state, matrix remodeling, angiogenic support, and fibrosis risk, but persistent mechanical memory in these populations still requires direct testing. FA, focal adhesion; LINC, linker of nucleoskeleton and cytoskeleton; BV/TV, bone volume fraction.

Mechanical effects on cells do not necessarily disappear after the stimulus is removed. Loading, matrix stiffness, and geometric confinement can induce persistent changes in cell structure and gene expression in multiple cell types [11] (Figure 3). Bone regeneration proceeds through temporally coordinated, but not necessarily synchronous, inflammatory, vascular, stromal, and osteogenic events [16, 17, 35]. After bone injury or scaffold implantation, immune cells contribute to debris clearance, inflammatory resolution, and foreign‐body responses; endothelial and stromal cells support tissue ingrowth; and stem/progenitor cells participate in osteogenic differentiation and matrix mineralization [16, 17]. Prior mechanical cues may therefore bias progenitor‐cell fate while mechanically conditioning immune and stromal states that influence the regenerative niche [18, 19]. Because persistence after cue removal has been established most clearly in stem/progenitor systems, the following subsections distinguish direct evidence of cellular mechanical memory from evidence of immune or stromal mechanosensing.

### 3.1 Cytoskeletal Remodeling and Focal Adhesions

Mechanical loading and matrix stiffness can produce lasting changes in actin organization, cell shape, and cell–matrix adhesions. On rigid substrates, MSCs form pronounced stress fibers and mature focal adhesions; after transfer to a compliant matrix, the retained state depends on priming duration, with short exposure remaining reversible and a sufficient mechanical dose producing persistent signaling [10]. YAP is a useful readout of retained stiffness history, although it should not be treated as the memory mechanism by itself. Matrix elasticity can direct lineage specification [36], and stiff priming can preserve nuclear YAP together with RUNX2 activity after transfer to a softer matrix [10]. Defined N‐cadherin ligation, however, can return YAP to the cytoplasm and reduce the effect of earlier stiffness exposure [37]. Thus, this memory is not fixed: its strength depends on the dose and duration of the original cue and can be weakened by later adhesive or mechanical conditions.

### 3.2 Nuclear Deformation and Chromatin Remodeling

Mechanical forces transmitted through the cytoskeleton can deform the nuclear envelope, reorganize chromatin, and change genomic accessibility [12, 38]. Histone acetylation and methylation offer one route by which these inputs can leave a more persistent transcriptional state [39]. In MSCs, sustained stretch produced persistent changes in cell alignment, chromatin condensation, energy metabolism, and smooth‐muscle differentiation after the loading program [40]. In hMSCs exposed to stiff hydrogels, histone acetylation and chromatin organization became reversible or persistent according to exposure duration [13]. A related expansion study in primary chondrocytes showed that prolonged two‐dimensional culture was associated with persistent remodeling of trimethylation at lysine 9 of histone H3 and incomplete recovery of the native phenotype after transfer to three‐dimensional hydrogels [14]. These findings support careful control of ex vivo culture mechanics, while also showing that persistence must be specified for each cell type and mechanical history.

### 3.3 Mechanotransduction Pathways and Transcriptional Circuits

Mechanical memory can be stabilized by regulatory circuits that couple cytoskeletal state to gene expression. During stiff priming of MSCs, myocardin‐related transcription factor A responds acutely to actin polymerization, whereas microRNA‐21 (miR‐21) remains elevated after cue removal and preserves a fibrotic program [15]. Stiff matrices can also drive gradual nuclear exit and degradation of the mechanorepressor NK2 homeobox 5, producing a scar‐like state [41]. In dynamic hydrogel models, miR‐21 tracked the early substrate mechanics and could be manipulated to erase or resensitize osteogenic memory [42]. YAP/TAZ provides another dose‐sensitive integrator of prior stiffness [10, 37], while mathematical models suggest that feedback between mechanosensitive transcription and cytoskeletal tension can stabilize cell state [43]. Dynamic stiffening can also activate signaling pathways associated with twist family basic helix‐loop‐helix transcription factor 1, transforming growth factor beta, and YAP in epithelial systems, but the absence of retained memory across repeated soft–stiff cycles in that model identifies it as time‐dependent mechanotransduction rather than direct evidence of memory [44].

### 3.4 Transient Versus Long‐Term Cellular Mechanical Memory

Mechanical memory in bone‐relevant stem/progenitor systems spans a hierarchical temporal spectrum [11, 20]. Transient memory, emerging over hours to days, includes reversible changes in cytoskeletal architecture, focal adhesions, actomyosin tension, and YAP/TAZ localization [37]. Longer‐term memory can involve nuclear organization, chromatin accessibility, histone modifications, metabolism, and self‐supporting transcriptional programs [12, 13, 38, 40]. These states may bias cells toward regenerative osteogenesis or maladaptive fibrotic programs, but their persistence and functional consequence remain cell‐ and context‐dependent [15].

### 3.5 Mechanical Conditioning of the Regenerative Niche

Although the strongest cue‐removal evidence for persistent mechanical memory comes from stem/progenitor systems, immune and stromal cells also respond to the changing mechanics of bone repair. Substrate stiffness and interfacial rigidity can alter macrophage membrane deformation, cytoskeletal organization, foreign‐body responses, and repair‐related gene programs [18, 19]. These studies establish mechanosensing and repair‐program regulation, but they do not directly show that the macrophage state persists after the mechanical cue is removed. Stromal cells can further contract, deposit, and reorganize the extracellular matrix, creating self‐reinforcing mechanical feedback [45, 46]. Thus, immune and stromal mechanics should presently be described as history‐dependent niche conditioning, while persistent mechanical memory in these populations remains an important hypothesis to test directly.

### 3.6 Consequences for Progenitor‐Cell Fate and Regenerative Potential

Earlier mechanical signals can continue to influence later regenerative‐cell behavior. In hMSCs, sufficiently prolonged stiff priming can preserve YAP/TAZ–RUNX2 activity and an osteogenic bias after matrix softening [10]. In skeletal muscle stem cells, exposure to stiff hydrogels during the first three days of activation reduced the later proliferative progenitor pool, whereas Ras homolog family member A (RhoA) inhibition during that window prevented formation of the detrimental memory [47]. Dynamic‐niche studies in hMSCs similarly show that early substrate mechanics can bias later osteogenic differentiation [42]. However, direct evidence that MSCs primed by prior mechanical exposure improve bone volume or mechanical strength after delivery to load‐bearing bone defects remains insufficient; this translational step should be treated as a hypothesis rather than an established outcome.

### 3.7 Reversibility and Erasure of Memory

Not all mechanical memories are permanent. Their reversibility depends on cue magnitude and duration, the recovery environment, competing adhesion or biochemical signals, and the level at which memory is stored. Short‐lived responses can remain in rapidly remodeling focal adhesions, actomyosin tension, and YAP/TAZ localization, whereas longer or stronger priming can engage nuclear organization, chromatin remodeling, metabolism, and self‐reinforcing transcriptional programs. Reversibility should therefore be evaluated across explicitly defined timescales rather than treated as a binary material or cellular property [20].

Representative experiments illustrate this graded behavior. In hMSCs, culture on very stiff tissue‐culture plastic for 1, 3, 7, or 10 days followed by transfer to soft hydrogels presenting the arginine‐glycine‐aspartate motif produced a duration‐dependent YAP response: short priming was readily reversible, intermediate priming was partially persistent, and N‐cadherin ligation provided a competing cue that returned YAP to the cytoplasm [37]. Prolonged exposure of hMSCs to stiff hydrogels also produced persistent chromatin remodeling, with reversibility depending on the duration of stiff exposure [13]. Soft‐matrix inhibition of YAP/TAZ therefore represents a candidate reset strategy rather than an established bone‐regeneration treatment [48]. In skeletal muscle stem cells, stiffness memory was set during the first three days of activation, and RhoA inhibition during this window blocked the detrimental phenotype [47].

Taken together, cells involved in bone repair appear to retain a trace of the mechanical environments they have experienced. The strongest cue‐removal evidence still comes from stem and progenitor cells. In these systems, previous substrate stiffness or loading can alter proliferation, differentiation bias, and responsiveness to later signals. For bone‐regeneration studies, evidence from stem/progenitor cells has immediate implications. Mechanical preconditioning may improve the regenerative potential of implanted cells, but routine expansion on rigid culture plastic may also imprint a mechanical history before implantation. Immune and stromal cells add a second layer because their stiffness‐dependent responses can influence whether progenitor programs unfold in a vascularized repair niche or are restricted by inflammation, fibrosis, or poor tissue ingrowth. This niche view connects cellular memory back to scaffold design: scaffolds and implants create many of the mechanical histories that cells record, and cells then remodel those materials and the surrounding matrix in return. The next section examines this reciprocal relationship between material‐encoded mechanical memory and cell‐retained mechanical memory during bone regeneration.

## 4 Interplay Between Material and Cellular Memory

**FIGURE 4:**
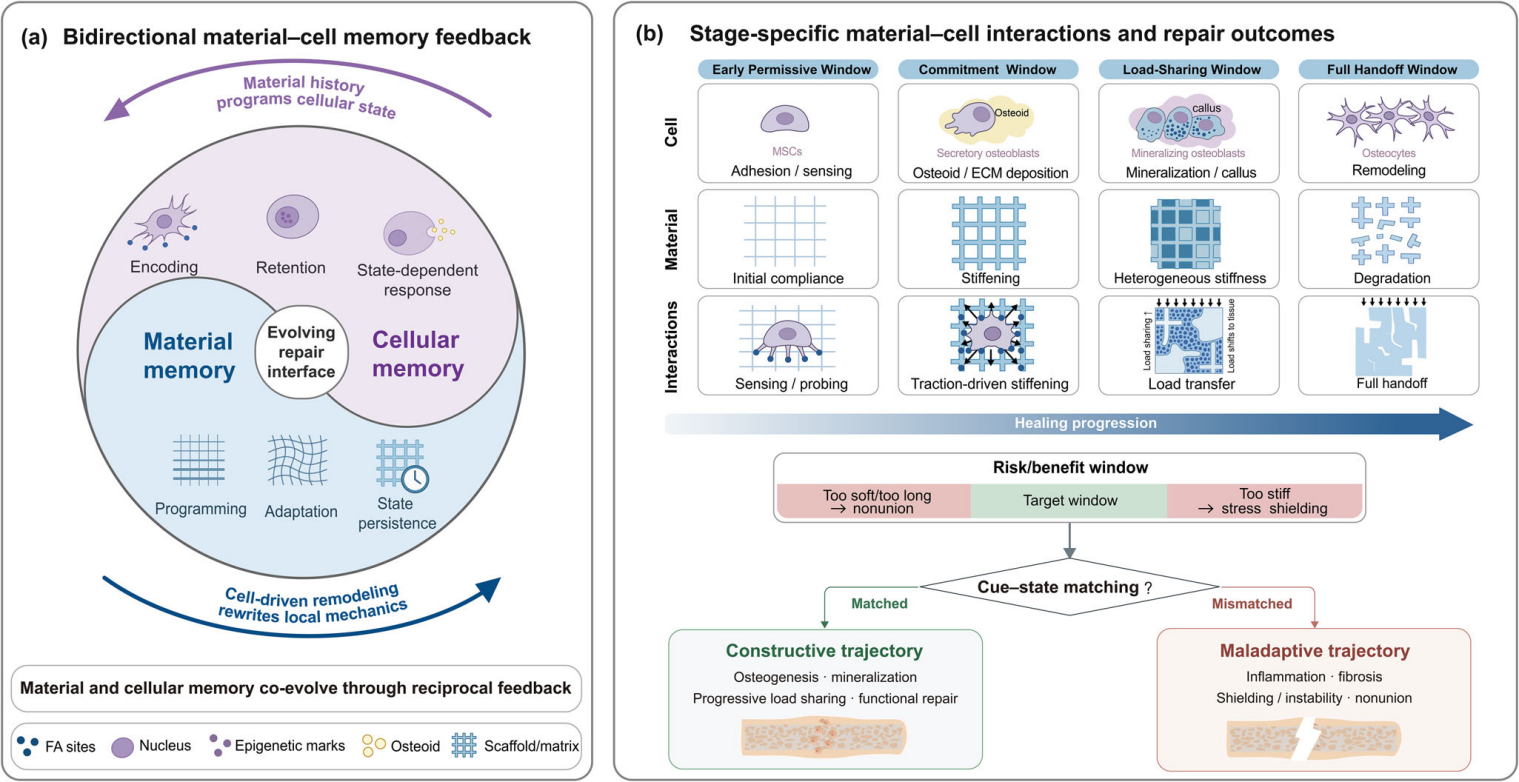
Bidirectional material‐cell memory feedback and stage‐specific repair outcomes. (a) At the repair interface, material memory and cellular memory influence one another. Scaffold stiffness, architecture, degradation, and adaptive mechanics can shape cell state, while matrix deposition, contraction, mineralization, and remodeling by cells can change the local mechanics that the material and tissue experience. This feedback can support repair or reinforce maladaptive states. (b) Stage‐specific material‐cell interactions should be matched to the evolving healing state. At the start of repair, the scaffold should remain compliant enough for infiltrating cells to adhere, sense, and probe the local matrix. The commitment window is where timing becomes critical: stiffening and traction‐driven feedback need to arrive while cells can still translate these cues into osteoid and early extracellular matrix deposition. As mineralization progresses, the callus becomes mechanically heterogeneous, and load transfer begins to matter more than stiffness alone. By the full handoff stage, scaffold degradation and tissue remodeling should shift mechanical function from the construct to the regenerate. When the cue arrives too early, too late, or at the wrong magnitude, the response may shift toward inflammation, fibrosis, stress shielding, instability, or nonunion.

Bone regeneration can be understood as a continuous interaction between the extracellular environment and the cells that reside within it. When both the material environment and the cells themselves retain forms of mechanical memory, this interaction begins to resemble a type of mechanical intelligence. In such a setting, past mechanical states influence later responses through a bidirectional feedback process (Figure 4). In the sections that follow, we outline key aspects of this interaction and discuss how they shape the course of bone healing.

### 4.1 Materials Programming Cell Memory

The key design question is not only what stiffness a scaffold should have, but when that stiffness should change relative to cellular fate decisions. If the repair environment becomes too stiff before cell infiltration and early matrix formation, local strain may be reduced, and stress shielding may occur. If it remains too compliant after reparative tissue begins to mature, the construct may fail to provide the mechanical support needed for mineralization and load‐bearing repair, increasing the risk of nonunion (Figure 4a). Scaffolds with material mechanical memory provide a way to manage this timing problem by delivering a sequence of mechanical cues rather than a fixed mechanical state. In a gradually stiffening hydrogel, MSCs first encounter a relatively soft matrix that supports spreading and proliferation; as the matrix stiffens, the same cells receive a later mechanical cue that promotes osteogenic differentiation [31]. When this transition overlaps with the relevant fate‐decision window, MSCs can retain the short mechanical episode as an osteogenic bias. In this sense, time‐dependent stiffening acts as material memory that is translated into cellular memory of lineage commitment.

Related ideas have also been explored in vivo using a two‐stage metamaterial scaffold with programmable mechanical behavior [9]. In that study, the scaffold decoupled low initial stiffness from later load‐bearing capacity: it exhibited an effective modulus of approximately 13 MPa under the early deformation regime and then became stiffer as strain increased. In a rabbit critical‐sized bone defect model, this adaptive design was reported to induce > 2% callus strain, which was associated with calcium‐channel‐related mechanosensing, hypoxia‐inducible factor 1 alpha signaling, and enhanced osteogenic and angiogenic gene expression. After four weeks, the two‐stage metamaterial scaffold increased the new bone fraction by 44% and 498% compared with conventional scaffolds with effective moduli of 500 and 13 MPa, respectively. This example suggests that programmed changes in scaffold mechanics can shape the mechanical signals perceived by repair cells and produce lasting effects on gene expression and bone healing.

### 4.2 Cells Modify Material Properties

Cells not only respond to scaffold mechanics but also change them. Through matrix remodeling, cells can leave lasting mechanical changes in the surrounding material [45]. A similar process happens during natural bone repair. In this process, osteoblasts deposit extracellular matrix. This matrix then gradually mineralizes and makes the callus stiffer as healing moves forward [49]. This increase in stiffness then affects cells again and can further support osteogenic activity [9]. Cells can also change local compliance by contracting the matrix or by degrading material components.

This idea may also help scaffold design. Cell‐generated forces could be used to change scaffold mechanics rather than merely treating scaffold mechanics as cues sensed by cells. Force‐triggered hydrogels have demonstrated rapid on‐demand microstructure growth at the material level [50], while synthetic multi‐dynamic hydrogels can combine stress stiffening with stress relaxation [51]. In cell‐remodeled collagen, stiff‐primed cells generate greater forces, align and tension fibers, and transfer mechanical history to the matrix, facilitating subsequent invasion [46]. These examples support the technical plausibility of cell‐to‐material feedback, although direct validation in bone regeneration remains limited.

A similar process may also operate in bone tissue engineering. If MSCs on a scaffold begin to mineralize one region, the associated local stiffening may encourage neighboring MSCs to undergo osteogenic differentiation, so that the response gradually extends beyond the initial site. This possibility suggests that scaffold design should take cell‐driven remodeling into account. Even when a scaffold starts out mechanically and structurally uniform, cellular activity may gradually create local differences in stiffness and porosity, giving rise to spatial heterogeneity that influences cell behavior throughout the regenerating tissue.

### 4.3 Time Scales and the Sequencing of Memory

A scaffold may stiffen, degrade, mineralize, or transfer load over days to weeks, but cells can respond to stiffness, strain, and confinement within minutes to days [7, 20, 31, 47]. This difference in time scale is central to scaffold design. If a fixation construct unloads the defect too early, cells may lose the strain signals needed before the tissue has gained competence [52, 53, 54]. If the construct remains too compliant, the defect may not receive the mechanical stimulus required for matrix maturation and load‐bearing repair [7, 31, 52, 53]. These observations suggest that temporal matching should begin with a permissive but mechanically stable environment for cell infiltration and vascularization, then provide osteogenic cues during cellular commitment windows, and gradually increase load sharing as matrix deposition and mineralization improve tissue competence. Because defect size, fixation stability, scaffold architecture, loading history, and patient background shift these windows, they should be treated as stage‐specific design windows rather than universal stiffness or time thresholds [20, 52, 53, 55].

This consideration makes temporal matching a design variable. From the cell side, relevant parameters include the duration and magnitude of mechanical priming, the timing of fate‐decision windows, and whether the mechanically induced state remains cytoskeletal and reversible or becomes stabilized through nuclear and transcriptional remodeling [12, 14, 20, 38, 40, 47, 56]. At the material and system levels, these cellular requirements can be translated into the onset and rate of stiffening or load transfer, local strain magnitude and frequency, stress‐relaxation behavior, degradation rate, and the capacity for on‐demand activation or reversal [7, 20, 31, 51]. Thus, material memory should be specified not only by its initial modulus or final strength, but also by the time course through which it exposes cells to mechanical information [7, 20, 31, 47].

Several design strategies follow from this principle. First, an early permissive phase should preserve construct stability while maintaining sufficient compliance, viscoelastic relaxation, and interconnected porosity to support cell infiltration, angiogenesis, and provisional matrix formation [7, 51, 57, 58]. Second, a transition phase can deliver osteogenic cues through timed crosslinking, mineralization‐coupled stiffening, shape‐memory recovery, force‐responsive linkers, or degradation‐mediated load transfer [6, 7, 31, 50]. The appropriate window remains context‐dependent: early stiffening favored osteogenic differentiation in a two‐dimensional system [30], whereas later stiffening after cell spreading enhanced osteogenesis in a three‐dimensional sequentially crosslinked hydrogel [31]. Third, late‐stage maturation can be supported by progressive load sharing, controlled micromotion, or fixation dynamization as tissue competence increases [52, 53, 55].

In practice, these windows should be adjustable rather than fixed. In one rat segmental‐defect model, delaying ambulatory loading until week 4 increased bone formation compared with immediate loading [59], whereas a recent near‐critical defect study found that resistance rehabilitation initiated one week after injury improved bone formation and functional recovery [52]. These studies indicate that loading windows are model‐ and indication‐dependent. Defect size, fixation stiffness, loading magnitude, and biological readiness can shift the point at which mechanical stimulation becomes beneficial. The same logic applies to dynamization: reducing construct stiffness or increasing interfragmentary motion may support callus formation in selected cases, but only after the tissue can tolerate the added motion [55]. Implant strain, callus stiffness, serial imaging, and wearable loading data could help identify this transition [52, 53, 60, 61]. Future scaffolds and implants may therefore incorporate feedback‐controlled strategies that adjust stiffness, micromotion, or rehabilitation intensity according to the evolving biological and mechanical state of the repair site. In this way, the local mechanical environment could be coordinated with cellular decision windows rather than following a predetermined material trajectory [20, 52, 61].

### 4.4 Bidirectional Mechanical Intelligence and Divergent Repair Outcomes

The preceding discussion defines when scaffold mechanics should change. A complementary design question is what type of feedback those changes initiate. When scaffold evolution, cellular state, and load transfer are appropriately coordinated, cell‐mediated matrix deposition and mineralization progressively enhance tissue competence, allowing more load to be transferred from the construct to the regenerate and supporting further maturation [9, 31, 49, 52]. The relevant design target is therefore not a one‐way stiffness program, but a constructive trajectory in which scaffold and tissue mechanics co‐evolve.

The same feedback can become maladaptive. A mechanically advanced but biologically immature environment may suppress osteogenic progression or prematurely transfer load away from regenerating tissue. Sustained high stiffness may reinforce contractile fibroblast states and self‐amplifying matrix remodeling [45, 62, 63]. Excessive interfacial stress or unstable force transmission can additionally activate mechanically driven foreign‐body responses [18, 64]. These responses can promote matrix contraction, inflammatory myeloid activation, and fibrous encapsulation, thereby compromising osseointegration and interface stability [17, 18, 64].

Based on this distinction, we propose that bidirectional feedback should be designed according to direction, gain, and reversibility. Direction describes whether feedback favors osteogenesis, vascularization, and progressive load sharing or promotes inflammation, fibrosis, and interfacial instability. Gain describes the extent of amplification: moderate feedback may reinforce matrix deposition and callus stiffening, whereas excessive feedback may produce self‐reinforcing stiffening, contraction, or fibrous encapsulation [9, 31, 45, 49, 52, 62, 63]. Reversibility describes whether the system can relax, degrade, dissipate stress, or redistribute load when maladaptive responses emerge. Compliant interfaces, viscoelastic damping, graded stiffness, and adaptive coatings may help control this feedback [5, 7, 23, 51]. Their biological purpose would be to guide mechanosensing toward pro‐resolving and tissue‐reparative programs rather than simply suppress macrophage activation [18, 19].

From this perspective, the relevant design variable is not only the initial modulus of the scaffold but also how the scaffold, callus, fixation construct, and cells change one another over time. A useful repair system would preserve feedback that favors osteogenesis and load sharing, but it should also detect when the same feedback begins to favor shielding, instability, fibrosis, or nonunion. This is why monitoring and adjustment are needed, and it leads to the discussion in Section 5 on sensing, prediction, stage‐specific mechanical dosing, and actuation.

## 5 Translating Mechanical Intelligence Into Bone Mechanomedicine

**FIGURE 5:**
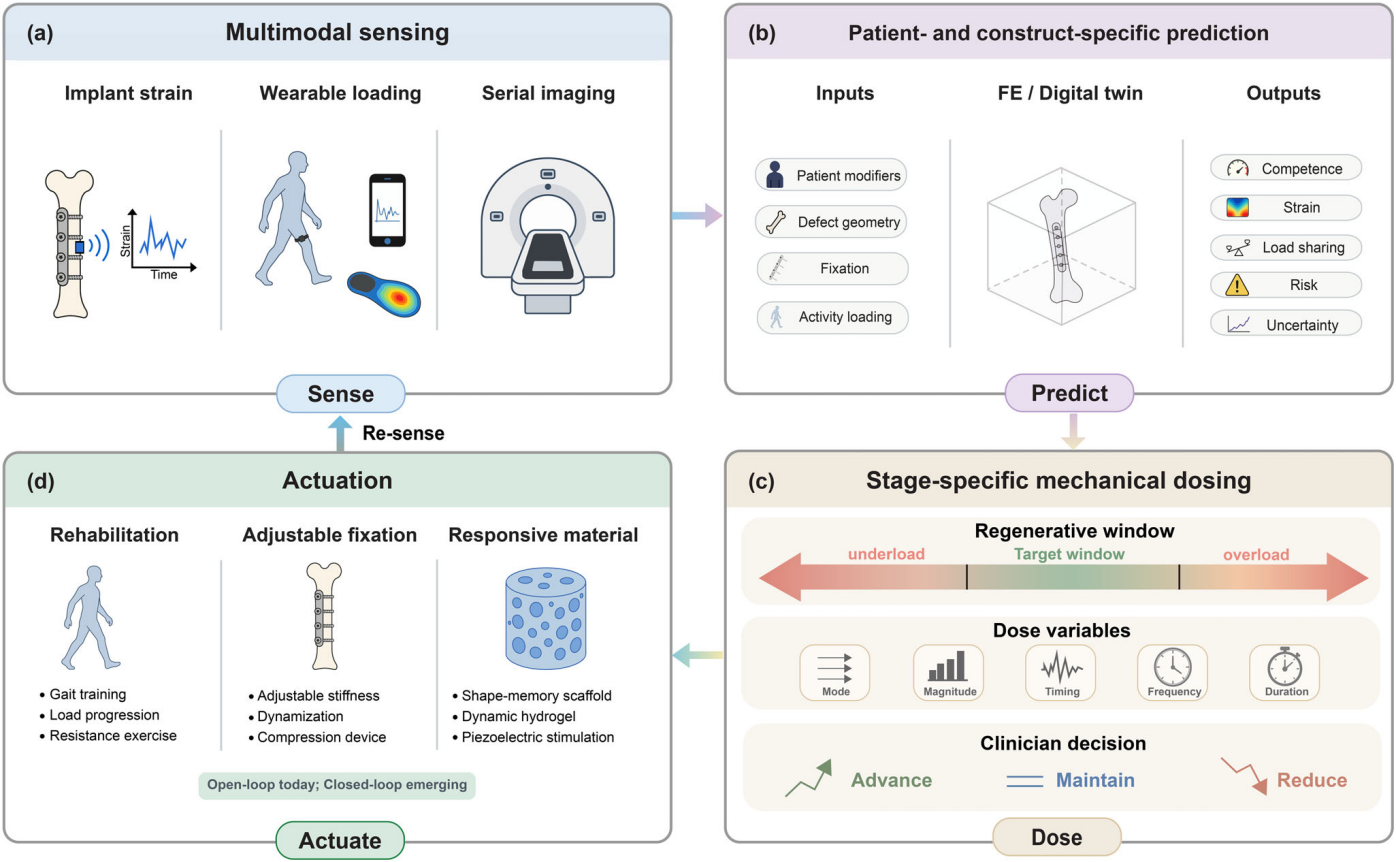
Closed‐loop bone mechanomedicine for patient‐specific bone repair. (a) Multimodal sensing combines implant strain measurements, wearable loading data, and serial imaging to monitor the evolving mechanical and structural state of the repair tissue. (b) Patient‐ and construct‐specific prediction integrates patient factors, defect geometry, fixation configuration, and activity‐derived loading into a digital twin or finite‐element model to estimate tissue competence, local strain, load sharing, healing risk, and uncertainty. (c) Stage‐specific mechanical dosing translates these estimates into clinically interpretable loading windows and dose variables, including loading mode, magnitude, timing, frequency, duration, and spatial distribution. The recommended action may be to advance, maintain, or reduce mechanical loading. (d) Actuation implements the prescription through rehabilitation, adjustable fixation, or responsive material systems. Repeated sensing closes the loop, but the near‐term goal is supervised, auditable decision support rather than fully autonomous control. FE, finite element.

For clinical use, the idea of mechanical intelligence has to be turned into decisions that a surgeon or rehabilitation team can actually use. The practical questions are concrete: whether healing is progressing mechanically, whether the current loading environment is regenerative or disruptive, whether and when fixation should be dynamized, and how rehabilitation should be adjusted as tissue competence develops. Addressing these questions requires a closed‐loop workflow in which the evolving mechanical state is measured, interpreted with patient‐ and construct‐specific models, converted into a stage‐specific mechanical dose, and delivered through rehabilitation, fixation adjustment, or material‐level actuation (Figure 5). The intended endpoint is clinician‐supervised decision support, not autonomous replacement of clinical judgment.

### 5.1 Sensing the Evolving Mechanical State

Implant‐based sensing provides a direct indication of how load is redistributed between fixation hardware and regenerating tissue. Instrumented fixation plates can record bending strain, while wireless and battery‐free systems are beginning to support longer‐term monitoring during daily activity [65, 66]. Under comparable loading conditions, declining implant strain can indicate increasing tissue competence as the callus carries more load. Because implant strain is also affected by activity, gait, fixation geometry, and sensor position, it should be interpreted with the loading context rather than as a stand‐alone marker.

Wearable systems provide a complementary view of the mechanical loading generated by patient activity. Ground‐reaction‐force or pressure‐sensing insoles can quantify loading distribution and gait‐related changes during rehabilitation [60]. Imaging provides structural information about defect geometry, callus formation, mineralization, and tissue continuity. These measurements operate at different scales: implants report construct‐level load transfer, wearables estimate external loading behavior, and imaging characterises structural progression. None directly reveals the complete mechanical environment within a heterogeneous defect, providing the rationale for multimodal predictive models.

### 5.2 Predicting Tissue Competence and Healing Risk

Patient‐ and construct‐specific models can integrate defect geometry, fixation configuration, activity‐derived loading, and tissue assumptions to estimate local strain, implant stress, and interfragmentary mechanics [53, 61]. Repeated implant, wearable, or imaging measurements could, in principle, update these estimates as healing progresses, but a continuously updated patient‐specific fracture twin has not yet been prospectively validated. The near‐term aim is therefore to determine whether the estimated trajectory is consistent with increasing mechanical competence or suggests delayed healing.

This interpretation must account for spatial heterogeneity. Spatial analyses of fracture repair show that high‐ and low‐strain regions within the same defect can be associated with distinct transcriptional programs [67]. Prediction should therefore connect patient‐level loading with construct mechanics and local tissue responses. Clinically useful outputs may include estimated load ranges, changes in healing risk, readiness for dynamization, and whether rehabilitation should be maintained, advanced, or reduced. These outputs are better presented as trends with uncertainty bounds than as a single deterministic forecast.

### 5.3 Stage‐Specific Mechanical Dosing

Once the mechanical state has been interpreted, it must be translated into a dose that can be delivered safely. Mechanical dose includes not only force or strain magnitude, but also loading mode, frequency, duration, spatial distribution, and timing relative to biological readiness. Section 4 established why these variables should be matched to cellular decision windows. At the clinical level, the corresponding task is to define stage‐specific operating ranges and progression criteria rather than a single loading threshold. Key design variables for stage‐specific mechanical dosing and their translational implications are summarized in Table 2.

**TABLE 2:** Design variables for stage‐specific mechanical dosing during bone repair.

Variable	Design consideration	Translational implication
Loading mode	Compression, tension or distraction, shear, cyclic loading, fluid‐flow‐mediated stimulation, and controlled micromotion can produce different tissue‐level and cellular responses.	Match loading mode to the intended repair stage, fixation geometry, and scaffold architecture rather than specifying load magnitude alone [54, 59].
Magnitude	Loading should generate sufficient local deformation to support mechanotransduction without destabilizing immature tissue or fixation.	Use construct‐specific strain or motion ranges rather than body‐weight percentage alone [52, 59].
Timing	The same mechanical dose may be beneficial after callus formation but disruptive before vascular and tissue readiness, depending on defect severity and fixation conditions.	Progress loading according to indicators of biological and mechanical competence rather than postoperative time alone [52, 58].
Frequency and duration	Intermittent loading may provide a regenerative stimulus while allowing recovery, whereas prolonged or repetitive overload may sustain inflammation or tissue damage.	Specify loading bouts, repetition frequency, and recovery periods in addition to peak load [52, 54].
Spatial distribution	Heterogeneous defects expose different regions and cell populations to different strain states.	Use fixation design, scaffold architecture, or rehabilitation strategy to avoid local overload and mechanically inactive regions [53, 67].
Progression and withdrawal	Mechanical dose should increase as tissue competence develops and be reduced when adverse mechanical, inflammatory, or functional responses emerge.	Define criteria for advancing, maintaining, or reducing rehabilitation and for adjusting construct stiffness [52, 55].

In practice, a proposed rehabilitation progression may move from protected weight bearing, pain‐limited range‐of‐motion exercise, and gentle muscle activation toward gait retraining, balance work, and strengthening as callus competence develops. Higher‐load exercises and return‐to‐activity tasks would follow only after adequate mechanical and clinical evidence of progression [52, 54, 59]. This sequence is a translational framework rather than a validated universal protocol and must be adapted to fracture site, fixation, comorbidity, pain, and functional status.

Experimental evidence illustrates how sensing can support such adjustment. In a rat segmental femoral‐defect model, resistance exercise increased defect strain by approximately 44% during the first two weeks and was associated with enhanced bone formation and recovery of mechanical function [52]. Real‐time strain measurement and finite‐element analysis made it possible to quantify the mechanical dose that was actually delivered, instead of estimating it from the exercise protocol alone. For translation, the harder problem is to define strain or motion ranges that are acceptable for a given defect, fixation construct, and patient activity. Rehabilitation should therefore not progress simply because enough postoperative time has passed. It should progress when the repair tissue shows evidence that more load is likely to support healing rather than disrupt it.

### 5.4 Actuating the Mechanical Prescription

Actuation is the point at which an interpreted mechanical state is turned into an intervention at the patient, construct, or material level. At the patient level, this may involve adjusting weight‐bearing progression, gait retraining, resistance exercise, movement frequency, loading duration, or functional task training. Sensor‐informed recommendations can help clinicians judge whether loading should be advanced, maintained, or temporarily reduced, while leaving room for pain, muscle function, comorbidities, radiographic progression, adherence, and the overall clinical context.

At the construct level, the adjustable variable is often fixation: stiffness, interfragmentary motion, and the proportion of load transferred to the regenerate can all be changed through adjustable fixation or dynamization [68]. Shape‐memory alloy devices provide a limited clinical precedent. NiTi staples, for example, can maintain compression across selected fusion or osteotomy sites through temperature‐dependent shape recovery [69]. Future programmable fixation systems may modify mechanical support according to evidence of callus maturation, but most current devices are still manually adjusted or follow a predefined program rather than responding autonomously.

At the material level, shape‐memory scaffolds, dynamically crosslinked hydrogels, degradable load‐sharing structures, and force‐responsive linkers can alter defect conformity, stiffness, stress relaxation, or load transfer over time [6, 7]. Such systems can generate spatially and temporally patterned mechanical outputs. Their biological value, however, depends on whether the programmed transition occurs at the appropriate stage of repair. A material response that is fixed in advance should therefore be distinguished from a closed loop, because it is not updated according to the state of the regenerating tissue.

Because bone itself exhibits electromechanical behavior [70], piezoelectric scaffolds offer a way to convert physiological deformation into local electrical signals. A periosteum‐bone‐mimicking piezoelectric bilayer, for example, has been reported to support neurovascularized bone regeneration through self‐powered electrical stimulation [71]. Piezoelectric hydrogels and related constructs may also couple patient movement with local electromechanical signaling [72]. These approaches broaden actuation beyond stiffness and load transfer, but their dose‐response relationships, long‐term stability, and safety still require systematic evaluation.

Most actuation strategies discussed above should still be regarded as open‐loop. In rehabilitation, loading is generally changed after periodic assessment; in programmable fixation, the construct follows a predetermined schedule; and in responsive materials, the output is governed by intrinsic material rules. A genuinely closed‐loop actuator would modify its output according to repeated measurements of the healing state. Keeping this boundary clear is important because otherwise responsive or programmable systems may be mistaken for true feedback‐controlled technologies.

### 5.5 Clinical Maturity and Implementation Barriers

The components of this framework are at different stages of development. Adjustable fixation, staged rehabilitation, and NiTi compression devices already provide clinical precedents for modifying mechanical conditions during healing. Implantable strain sensing and wearable loading assessment are nearer to pilot or early translational use. Patient‐specific prediction, sensor‐guided dynamization, and responsive scaffold actuation remain largely preclinical, and a fully integrated autonomous system remains speculative.

Several barriers remain before closed‐loop bone mechanomedicine can become routine. Mechanical readouts need to be standardized across implant geometries and patient activities, and stage‐specific operating ranges need validation in different defects, fixation conditions, anatomical sites, and patient groups. Sensors and responsive materials must also remain stable without compromising fixation or tissue integration. Finally, model outputs have to be interpretable in the clinical workflow in which they will be used. Regulatory pathways will also need to account for systems in which the therapeutic output changes over time.

The near‐term objective is therefore not a fully autonomous implant, but a clinically interpretable loop in which mechanical measurements inform patient‐specific decisions, clinicians retain oversight, and intervention is updated as healing progresses. Such a framework would translate mechanical intelligence from a material property or biological concept into an operational strategy for bone mechanomedicine.

## 6 Future Outlook: Toward Healing‐State‐Aware and Clinically Validated Bone Mechanomedicine

The technologies discussed above have reached markedly different levels of maturity. Responsive scaffolds, implantable sensing, patient‐specific modelling, and machine‐learning prediction have each been demonstrated in selected experimental or early clinical settings, whereas their integration into autonomous bone‐healing systems has not. Future work should therefore distinguish experimentally validated components from plausible system architectures and from research hypotheses.

### 6.1 Requirements for Healing‐State‐Aware Material Adaptation

The next step beyond responsive biomaterials is healing‐state‐aware adaptation, in which a change in material behavior is conditional on the evolving state of repair rather than being triggered indiscriminately by time, hydration, or a single loading event. Several enabling components have already been demonstrated experimentally. Water‐responsive silk‐fibroin/magnesium scaffolds can recover their shape, conform to irregular defects, and support cranial bone regeneration in rats [6]. Macroporous hydrogels with mechanically reinforced pore shells and tunable degradation can provide spatially heterogeneous and temporally programmed cues, with bone‐regenerative efficacy demonstrated in rabbit and porcine defects [7]. Synthetic multi‐dynamic hydrogels can also combine stress stiffening with stress relaxation [51]. These systems validate shape adaptation, programmed mechanical evolution, and nonlinear load response, but they do not yet determine whether the tissue has reached a specific inflammatory, vascular, or load‐bearing state before changing their output.

A healing‐state‐aware material would therefore require four linked capabilities: a measurable proxy for healing state; a decision rule that distinguishes under‐stimulation, regenerative loading window, and overload; bounded adaptation of a clinically relevant material property; and an appropriate spatial range, time constant, reversibility, and fail‐safe state. Mechano‐adaptive meta‐gels provide an engineering proof of principle for this architecture by enabling strain threshold‐initiated signal propagation and nanofiber assembly, which increased the hydrogel modulus approximately sevenfold [73]. However, this platform was not designed for implantation and has not been validated for biocompatibility, cyclic skeletal loading, or bone repair.

It is therefore reasonable to propose, but not yet claim as an established technology, a workflow in which a mechanically instrumented scaffold or fixation construct detects changes in defect strain or load sharing, applies a validated rule, and triggers bounded local stiffening, relaxation, degradation, or compression. The individual sensing, processing, and actuation functions are technically compatible, yet their integration has not been validated as a closed‐loop bone‐regeneration system. A still more ambitious concept—a distributed scaffold that autonomously distinguishes insufficient loading from osteogenic stimulation and overload, adapts bidirectionally in different defect regions, and resets as tissue remodels—remains a research hypothesis. Progress should therefore be judged not by responsiveness alone, but by whether adaptation is linked to healing state, quantitatively bounded, reversible where necessary, and shown to improve healing relative to a predefined or open‐loop material program.

### 6.2 From Multimodal Measurements to Validated Decision Support

Digital twins and artificial intelligence are most useful when they connect measurements that describe different parts of the healing problem rather than being presented as a generic “cognitive core”. Relevant inputs are already available at several scales. Battery‐free osseosurface sensors have enabled long‐term wireless bone‐strain recording in freely moving sheep and serial measurement of a time‐dependent strain pattern during fracture repair [66]. Wearable insoles that measure ground reaction force have also been evaluated retrospectively in 25 patients with lower‐extremity fractures; an interpretable logistic‐regression classifier assigned gait steps to time‐to‐healing categories with a mean leave‐one‐patient‐out accuracy of approximately 76% [60]. At the modelling level, a 2025 orthopedic‐trauma study combined patient‐specific imaging, motion capture, musculoskeletal modelling, and finite‐element analysis in five nonunion revision cases to compare predicted implant stresses and fracture‐region strains before and after alternative surgical strategies [61]. These studies validate individual measurement and modelling components, but none demonstrates a continuously updated digital twin that has prospectively improved rehabilitation decisions or healing outcomes.

A concrete near‐term workflow could use postoperative computed tomography to define defect and fixation geometry, serial radiographs to track structural progression, implant‐ or bone‐surface strain to estimate changing load sharing, and wearable gait or ground‐reaction‐force data to define patient‐specific loading boundary conditions. These measurements could update a finite‐element twin that estimates local defect strain, interfragmentary motion, and callus stiffness. To reduce the computational cost of repeatedly solving the full model, a mechanobiology‐guided surrogate algorithm could be used. One recent study trained seven machine‐learning approaches on 648 trajectories generated by a previously validated 21‐day finite‐element model of rodent femoral fracture healing. A sequence‐to‐sequence long short‐term memory network performed best when predicting regional callus stiffness and total strain energy, including tests on 100 unseen interpolated scenarios; however, forecast accuracy declined as extrapolation extended beyond the observed time window [74]. Such forecasts could, in principle, be used to compare whether loading should be maintained, advanced, or reduced at a given healing stage, although this use remains to be prospectively tested. The model therefore provides a specific AI example for rapid mechanics‐informed forecasting, but remains a simulation‐based surrogate rather than a patient‐validated rehabilitation algorithm.

The output of such a system should initially be decision support, not autonomous control. For example, it could report whether estimated tissue competence is increasing as expected, provide uncertainty‐bounded strain or load‐sharing ranges, and indicate whether protected loading should be maintained, advanced, or reduced. Validation must occur at the patient rather than signal or simulation level: temporally separated and external cohorts should test calibration and discrimination; subgroup analyses should assess fracture site, fixation type, and patient modifiers; and prospective silent‐mode studies should precede trials in which recommendations influence care. Risk of bias and applicability should be assessed using contemporary prediction‐model standards such as the updated Prediction model Risk Of Bias ASsessment Tool for regression and artificial intelligence methods (PROBAST+AI) [75]. Physics‐informed neural networks could eventually impose equilibrium, constitutive, or healing constraints during model updating, while reinforcement learning or Bayesian optimization could search among staged loading policies. To date, patient‐level evidence showing that these algorithms can select rehabilitation timing and improve fracture outcomes remains lacking. Their use for weight‐bearing progression therefore remains a research hypothesis, as does federated learning for cross‐center model updating.

### 6.3 Patient‐Specific Modifiers of Mechanical Memory and Loading Windows

Personalized mechanotherapy should account for factors that alter both baseline tissue competence and the response to a given mechanical history. In strain‐matched tibial loading experiments, aged mice showed fewer loading‐induced protein changes than young‐adult mice and evidence of a delayed molecular response [76]. Sex and hormonal signaling also modify mechanoadaptation: osteocyte‐specific deletion of estrogen receptor‐β produced age‐, sex‐, and bone‐compartment‐dependent changes in the cortical anabolic response to two weeks of compressive loading [77]. Disease can further shift the relation between applied load and local tissue state. A calibrated fracture‐healing model predicted a larger mechanically unstable callus region in osteoporotic than non‐osteoporotic bone under the same load, with more severe impairment when osteoporosis and diabetes coexisted [78]. In diabetic mice, macrophage metabolic memory persisted after glucose normalization and continued to impair osteogenesis, angiogenesis, and fracture repair [79].

These findings indicate that age, sex, hormonal status, osteoporosis, and diabetes can modify mechanosensitivity, inflammatory persistence, repair capacity, or load tolerance. They do not yet show, however, how each factor changes the formation or erasure kinetics of mechanical memory itself. A universal osteogenic loading window should therefore not be assumed. Older or metabolically compromised patients may require lower initial loads, slower progression, and longer monitoring, whereas sex‐ and hormone‐associated effects may differ by skeletal site and cortical or cancellous compartment. Patient‐specific models should therefore treat these variables as factors that modify initial tissue properties, healing rate, and uncertainty. In clinical use, loading should advance according to measured tissue competence, rather than according to diagnosis or postoperative time alone.

### 6.4 Validation and Interdisciplinary Translation

Translation has to test the full measurement‐model‐decision‐actuation chain, not only isolated demonstrations of a responsive material, sensor, or prediction algorithm. Engineering verification should include sensor calibration and drift, material fatigue and biocompatibility, actuator reversibility and failure states, model uncertainty, and sensitivity to boundary conditions. Mechanistic and preclinical studies should then compare healing‐state‐aware or sensor‐guided systems with fixed, time‐programmed, and clinician‐adjusted controls. These studies should use repeated loading, relevant defect and fixation geometries, and, where feasible, both sexes, aged animals, and osteoporotic or diabetic conditions. Outcomes should extend beyond bone volume to vascularization, inflammation, interface stability, callus mechanics, functional loading, and failure or rescue events.

Clinical evaluation should proceed in stages. Retrospective and external validation should come first, followed by prospective silent‐mode testing in which recommendations are generated but do not yet influence care, and only then by clinician‐in‐the‐loop interventional trials. Prediction models should be tested for calibration, discrimination, clinical utility, subgroup performance, and dataset shift using frameworks such as PROBAST+AI [75]. Systems that update after deployment also require version control, traceability, predefined change limits, cybersecurity, and continuous monitoring. The FUTURE‐AI consensus similarly emphasizes fairness, universality, traceability, usability, robustness, and explainability across the healthcare‐AI lifecycle [80]. For mechanically intelligent systems, these requirements apply not only to software but also to the coupled sensor, implant, material, rehabilitation protocol, and human decision process.

No single discipline can validate this chain alone. Bone mechanobiologists need to define biologically meaningful state variables and loading windows; materials scientists and mechanical engineers need to test durability and bounded actuation; imaging and sensor specialists need to standardize longitudinal measurements; and computational scientists need to quantify uncertainty and model drift. Orthopedic surgeons, rehabilitation specialists, patients, trialists, regulators, and ethicists must then determine whether the recommendations are actionable, equitable, and clinically beneficial. The realistic near‐term goal is a supervised and auditable decision‐support system rather than a fully autonomous implant. Higher levels of automation should be considered only after such systems show reproducible benefit over fixed or open‐loop care.

## Statement on Use of Generative AI

During the preparation of this manuscript, the authors used ChatGPT 5.5 to improve the clarity and readability of the text, as well as to assist in the conceptualization of figure designs. All final illustrations were manually created by the authors. The tool was employed under human supervision, with each step undergoing rigorous quality control. All scientific content was thoroughly reviewed and edited by the authors, who take full responsibility for the final manuscript.

## Conflicts of Interest

The authors declare no conflicts of interest.

## Data Availability

Data sharing is not applicable to this article as no new data were created or analyzed in this study.
